# Supplementary material for: Extrusion-Based 3D Printing of Pharmaceuticals—Evaluating Polymer (Sodium Alginate, HPC, HPMC)-Based Ink’s Suitability by Investigating Rheology
Source: Micromachines (Basel). 2025 Jan 30;16(2):163. doi: 10.3390/mi16020163 (PMC11857113; doi:10.3390/mi16020163)
Supplement: Supplementary file 1 [file micromachines-16-00163-s001.zip › micromachines-3431348-supplementary.pdf]

Extrusion-Based 3D Printing of Pharmaceuticals—Evaluating Polymer (Sodium Alginate, HPC, HPMC)-Based Ink's Suitability by Investigating Rheology

Farzana Khan Rony [1,2], Georgia Kimbell [2], Toby R. Serrano [2], Destinee Clay [2], Shamsuddin Ilias [3] and Mohammad A Azad [2,*]

[1] Department of Applied Science and Technology, North Carolina A&T State University, Greensboro, NC 27411, USA; fkrony@aggies.ncat.edu
[2] Materials Science and Process Engineering (MSPE) Lab, Department of Chemical, Biological, and Bioengineering, North Carolina A&T State University, Greensboro, NC 27411, USA; glkimbell@aggies.ncat.edu (G.K.); tserrano@aggies.ncat.edu (T.R.S.); djclay@aggies.ncat.edu (D.C.)
[3] Department of Chemical, Biological, and Bioengineering, North Carolina A&T State University, Greensboro, NC 27411, USA; ilias@ncat.edu
* Correspondence: maazad@ncat.edu; Tel.: +1-336-285-3701; Fax: +1-336-334-7904.

Table S1. Recovery (%) information of various polymer-based inks was obtained from the Thixotropy test at different times.

Polymer and its concentration (%, w/w)	Recovery (%) at different times		
	**2.9 sec**	**63.8 sec**	**145 sec**
0.8% SA	2.89	11.48	18.32
1.6% SA	12.97	13.78	22.22
3.2% HPC H	42.68	27.11	31.79
4.8% HPC H	22.42	63.52	71.13
1.05% HPMC K100	81.65	96.93	97.18
1.05% HPMC K4	-	-	-
